# Vesico-Vaginal Fistula, No after Treatment

**Published:** 1874-06

**Authors:** M. Schuppert


*NEW ORLEANS MEDICAL AND SURGICAL JOURNAL—March,* 1874:

VESICO-VAGINAL FISTULA—*By M. Schuppert, M.D.*—* * * I have mentioned that the improvement in this operation dated from the enthusiasm of an American surgeon. It was the publication of a small pamphlet of Dr. Sims, with the high-sounding title, "Silver Sutures in Surgery the Greatest Achievement of the 19th Century." Now we know that the success of this operation is not due, does not depend upon the use of silver wire sutures, to which Sims mainly attributed his success, though I myself, like other American surgeons, am still using them—and I, from the fact that I find them to be handled in this operation far easier than sutures of any other material. Dr. Simon, in Heidelberg, Germany, and others, have proved to the satisfaction of every unprejudiced student, that with the use of silk as sutures the success is not a title smaller, but just as great, so that I may say, without fear of contradiction, the great improvement in the management of this disease rests with the means in our possession of gaining a better access to the parts to be operated upon—in other words, with a properly constructed speculum. The material of the sutures, whether of silk or metallic wire, is entirely indifferent (others, like Simpson, have given annealed iron wire even a preference to silver, in being cheaper, more malleable, and equally free from rusting). With a proper speculum, good paring, and coaptation of the borders of the fistula, we may be assured of success in performing this operation. We may dispense with everything else, including the catheter and the whole after treatment, without diminishing the probability of a successful issue. To accomplish the cure of a case of vesico-vaginal fistula, the after treatment has always been considered to be a very important part of it. The patient is kept in bed for many days in a very wearisome position; her bowels are confined with opium to exclude all intestinal movements; a catheter is, during the course of treatment, applied by the urethra, to keep the bladder empty, and to hinder the urine from coming in contact with the newly-united edges of the fistula; and finally, a certain diet is strenuously recommended. Of all the inimical agencies thought to stand in the way of a happy result, that most feared is the urine. To such an extent had the terror of that obnoxious fluid even affected me, that in my former operations I have thought it necessary to wash out the bladder during the first forty-eight hours after the sutures were applied, every half an hour. Was there no justification for this action? Had not even a great surgeon recommended, besides the use of the catheter by the urethra, the puncture of the bladder above the pubes, in order to keep the bladder entirely empty? Did not every surgeon of any experience in the operation speak of the permanent application of the catheter as the most important part of the after treatment? Did not Sims, the great apostle of the silver *wire* sutures, attribute a great deal of his succes to his "happy invention" of a properly shaped catheter? Dr. Emmet, in the latest work on vesico-vaginal fistula, still says: "To Sims' catheter we are greatly indebted for the success in this operation, as well as for much additional comfort to the patient. It should not touch the fundus of the bladder, and be nicely balanced in the urethra, and lie close up behind the pubis. A want of attention to this point will cause a failure of the operation; yet death may occur from the perforation of the bladder. The patient must lie the greater part of the time on the back, and, if possible, preserve this position until after the sutures have been removed. The catheter must be removed several times a day for the purpose of cleaning it. It is well to have two catheters, so that one may be introduced immediately upon the removal of the other. A sufficient quantity of opium should be administered daily to keep the bowels constipated until the sutures are removed. The catheter must be continued in use for a few days longer, and from the fourteenth to the twentieth day the patient may sit up."

Dr. N. Bozeman uses the elastic catheter, which he prefers to all others, on account of the comfort of the patient and "the ease," as he says, "with which it can be kept open without removal, simply by running a wire through it."

In the face of such authorities and precedents, it will probably be considered a bold innovation when I recommend not only the abolition of the catheter, of whatever material or shape it may be constructed, but also (the removal of the sutures excepted) all other parts of the after treatment.

